# The Columbia-suicide severity rating scale: validity and psychometric properties of an online Spanish-language version in a Mexican population sample

**DOI:** 10.3389/fpubh.2023.1157581

**Published:** 2023-09-05

**Authors:** Fernando Austria-Corrales, Alberto Jiménez-Tapia, Claudia Iveth Astudillo-García, Paulina Arenas-Landgrave, Tonatiuh Xochihua-Tlecuitl, Copytzy Cruz-Cruz, Leonor Rivera-Rivera, José Alberto Gómez-García, Bruma Palacios-Hernández, Berenice Pérez-Amezcua, Filiberto Toledano-Toledano, Jenelle Richards, Igor Galynker

**Affiliations:** ^1^Centro de Investigación en Salud Poblacional, Instituto Nacional de Salud Pública (INSP), Cuernavaca, Morelos, Mexico; ^2^Instituto Nacional de Psiquiatría Ramón de la Fuente Muñiz (INPRFM), Mexico City, Mexico; ^3^Facultad de Psicología, Universidad Nacional Autónoma de México, Mexico City, Mexico; ^4^Facultad de Ciencias, Universidad Nacional Autónoma de México, Mexico City, Mexico; ^5^Servicios de Atención Psiquiátrica (SAP). Secretaría de Salud, Mexico City, Mexico; ^6^Independent Researcher, Mexico City, Mexico; ^7^Centro de Investigación Transdisciplinar en Psicología, Universidad Autónoma del Estado de Morelos, Cuernavaca, Morelos, Mexico; ^8^Unidad de Investigación en Medicina Basada en Evidencias, Hospital Infantil de México Federico Gómez, Mexico City, Mexico; ^9^Unidad de Investigación Sociomédica, Instituto Nacional de Rehabilitación Luis Guillermo Ibarra Ibarra, Mexico City, Mexico; ^10^Dirección de Investigación y Diseminación del Conocimiento, Instituto Nacional de Ciencias e Innovación para la Formación de Comunidad Científica, INDEHUS, Mexico City, Mexico; ^11^Department of Psychology, Texas State University, San Marcos, TX, United States; ^12^Icahn School of Medicine at Mount Sinai, New York, NY, United States

**Keywords:** suicidal behavior, suicide risk, C-SSRS, validation study, risk assessment

## Abstract

The aim of this study was to evaluate the validity and psychometric properties in a Mexican sample of a Spanish-language online version of the Columbia-Suicide Severity Rating Scale (C-SSRS). Data were collected between May and October 2021 from 3,645 participants aged 18 years and over, who agreed to complete the questionnaire. Reliability analysis, confirmatory factor analysis (CFA), and psychometric properties were calculated using a two-parameter model. The results showed a reasonable level of reliability with a Cronbach’s alpha of 0.814, and evidence of unidimensionality, and construct validity for suicide risk at three risk levels: low, medium, and high. Analysis of the items suggests that they are consistent with the proposed theoretical model. Our results also demonstrate that the parameters are stable and able to efficiently discriminate individuals at high risk of suicide. We propose the use of this version of the C-SSRS in the Spanish-speaking population, since it is a multifactorial assessment of suicide risk and the inclusion of other clinical and risk factor assessments for a more comprehensive evaluation.

## 1. Introduction

Suicide is a global public mental health problem. Data show that 703,000 people died by suicide in 2019, making it one of the world’s leading causes of mortality; it produces more deaths than causes such as malaria, HIV/AIDS, breast cancer, wars, and homicide (1). The global age-standardized suicide rate is about 9 per 100,000 population, with variation among countries ranging from 2 to 80 deaths per 100,000 population, and it occurs mainly in low– and middle-income countries, where most of the world’s population lives. Data also show that suicide mortality in the Americas increased by 17% between 2000 and 2019 (1). In Mexico, the mortality rate from suicide in 2022 was 6.5 per 100,000 population, with the 15–29 age group having the highest risk (a rate of 16.2), making suicide the fourth leading cause of death in this group, exceeded only by violence, accidents, and COVID-19 (2).

The effects of the COVID-19 pandemic, while permeating the mental health of the entire population, have not had a uniform effect worldwide. For example, a study of suicide data recorded 9 to 15 months after the onset of the pandemic in 33 countries reported no evidence of an increase in the number of suicides in most of them; however, in middle- and low-income countries the data showed evidence of an increase (3). In the 32 states of Mexico, there was a differential impact on suicide deaths, suggesting that higher population density was associated with the increase in suicides in 2019, which highlights a need to improve access to primary care and mental health services to meet the needs of the population in emergency situations (4).

Every suicide represents an individual tragedy and has far-reaching effects on families and communities, so suicide must be approached from a public health perspective. This perspective should: (a) assess the magnitude of the problem, (b) examine differences in rates among groups and geographic regions, and (c) establish local, provincial/state, and national health priorities (5). The public health approach provides an understanding of the characteristics and interactions among factors that could contribute to improved surveillance, monitoring, and timely clinical care (6). Thus, early identification and timely intervention is critical for individuals at suicide risk, and a systematic screening process should be established (7).

In the context of monitoring and surveillance of health indicators, many tools are available for detecting suicidality and determining the level of risk, but their scope, advantages, and disadvantages are controversial. A systematic review found that there is no strong evidence that any tool is accurate enough to predict suicide with a sensitivity of 80% and a specificity of 50% (8). Given the current limitations in identifying individuals who may die by suicide, the bases of prevention are universal strategies combined with expertise in psychiatry and risk assessment (9). Although it has been reported that self-reported suicidal ideation (SI) may be a poor predictor of suicidal behavior (10–12), it remains the core of risk assessment, so research on culturally appropriate and reliable scales, such as the Columbia-Suicide Severity Rating Scale (C-SSRS), is essential.

The C-SSRS was developed as a semi-structured assessment instrument based on a clinical interview (13), and evaluates the presence, severity and frequency of suicide ideation and behavior; includes questions to explore the presence of ideation, the intensity of ideation, and suicidal behavior (including information on preparatory actions, as well as actual, interrupted, and aborted attempts). The CSSRS is a widely used method for screening and assessing suicide risk in clinical and research settings and for that reason requires proper validation. Still, aspects of the scale design and measurement model have received scant empirical investigation (14, 15). Knowledge about the construct validity of suicidal ideation severity is insufficient, particularly about the intrinsic properties of the items as consecutive indicators of suicide risk severity (14), and although the scale is available in more than 100 languages, there have been few evaluations outside of English-speaking populations (15). A literature search up to 2022 identified only two validation studies of the psychometric properties of the Spanish version of the C-SSRS, one study conducted with adult psychiatric outpatients, whose results showed weak internal consistency and convergent validity, but strong discriminant validity (16), and another study conducted with college students that reported evidence of validity and reliability (17). Both examined the multidimensional version of the 21-item interview.

In the present study we focus on the assessment of suicide risk severity as an important step for prevention, considering suicidal behavior as a unidimensional construct and considering the relative importance of each item as a measure of the underlying latent construct. The main objective of this study was thus to evaluate the validity and psychometric properties of an online Spanish-language version of the C-SSRS in a sample of Mexican adults aged 18 years and over.

## 2. Materials and methods

### 2.1. Method

This study was part of the multinational cross-sectional study “Effects of Quarantine on Degree of Emotional Distress During the COVID-19 Outbreak” (study no. GCO: 20–03543 IF: IF2644172), registered with the Mount Sinai Health System, United States. The study in Mexico was entitled “Evaluation of the Suicidal Crisis Syndrome (SCS) during the COVID-19 Pandemic, “and was approved by the Research Ethics Committee of the Instituto Nacional de Psiquiatría Ramón de la Fuente Muñiz (study no. CEI/C/059/2020).

### 2.2. Participants and procedure

Data for the study were collected using an electronic questionnaire distributed through the Qualtrics platform to a sample of adults from the general population. Invitations to participate were posted on social media (Facebook, Instagram, WhatsApp, Twitter), by email, and on the websites of various health and educational institutions. Participants were provided with a directory of support resources to which they could turn if required, both in the announcement inviting them to participate, and when they completed the instruments. The sample included 3,645 participants of both genders, who answered the questionnaire between May and October 2021. Ages ranged from 18 to 84 years (*M* = 35.51, *SD* = 12.24), and the majority were female (71%), single (55%), had a bachelor’s degree (62%), were health care personnel (50%), and were employed full-time (51%).

### 2.3. Study variables

#### 2.3.1. Columbia – suicide severity rating scale screener

The C-SSRS (13) assesses the severity and intensity of suicidal ideation and the occurrence of suicidal behavior during the person’s lifetime. This version of the scale is used on admission to clinical settings and in research to inquire about the respondent’s suicidal thoughts and behaviors in a face-to-face session. In the present study, a version of the scale with 12 items that can be answered dichotomously (yes/no) was used. In this version, two time periods are examined: once in a lifetime and in the last month.

The analysis included a conceptual review of the 12 items of the C-SSRS from the original protocol to adapt the short, self-administered version. The conceptual review involved two judges with expertise in mental health and suicide who independently analyzed each item on the scale. We selected six of the twelve items that best represented the spectrum of suicidal construct: ideation, suicide planning, and suicide attempt. Agreement on relevance, appropriateness, and severity was unanimous. These items are similar to those in the Spanish version of the C-SSRS, “Exploratory version – since last visit” (13), but in our version all items are used regardless of the response to the first two. Table 1 shows the comparisons between the two versions in terms of what was experienced in the last month. The Spanish version of the scale is included in the Supplementary Table S1.

**Table 1:** Comparison of the C-SSRS-exploratory version-recent (2008) and the items included in the current study.

C-SSRS-Exploratory Version-Recent			Current Study		
	Past month			Past month	
Ask questions 1 and 2	Yes	No	Answer all the questions	Yes	No
1. Have you wished you were dead or wished you could go to sleep and not wake up?	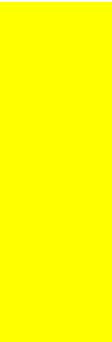		1. Have you thought (even momentarily) that you would be better off dead, wished you were dead, or felt like you needed to die?	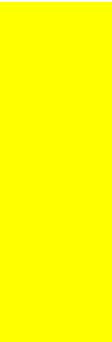	
2. Have you actually had any thoughts of killing yourself?	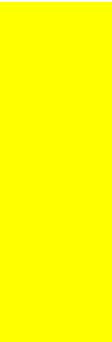		2. Have you thought (even momentarily) about harming, hurting, or injuring yourself with at least some intent or awareness that you may die as a result?	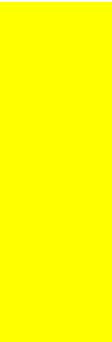	
If YES to 2, ask questions 3, 4, 5, and 6. If NO to 2, go directly to question 6.					
3. Have you been thinking about how you might do this?	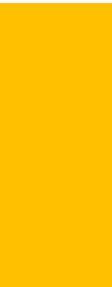		3. Have you had a plan (i.e., a place/date/timeframe) in mind to attempt suicide?	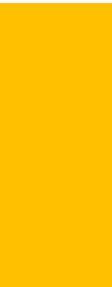	
4. Have you had these thoughts and had some intention of acting on them?	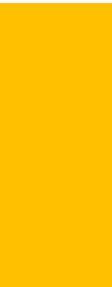		4. Have you taken any active steps to prepare for a suicide attempt in which you expected or intended to die?	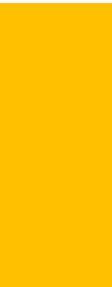	
5. Have you started to work out or worked out the details of how to kill yourself? Do you intend to carry out this plan?	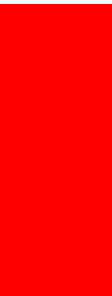		5. Have you started a suicide attempt, but then decided on your own to stop and did not finish the attempt?	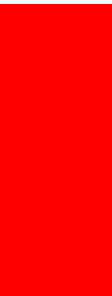	
6. Have you ever done anything, started to do anything, or prepared to do anything to end your life?	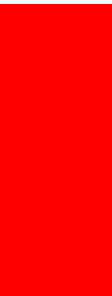		6. Have you started a suicide attempt, but then you were interrupted by someone else and did not finish the attempt?	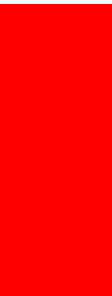	

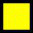
 Low Risk. 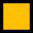 Medium Risk. 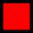 High Risk.

### 2.4. Data analysis

Psychometric testing was performed using a reliability analysis that included Cronbach’s alpha. A tetrachoric covariance matrix was then analyzed since the variables are dichotomous (18). Confirmation of unidimensionality assumptions and construct validity was performed with confirmatory factor analysis (CFA), using the Satorra-Bentler correction because the data lacked multivariate normality (19). The evaluation of the model included four fit indices: (I) the Comparative Fit Index (CFI), whose values range from 0 to 1 (a value of 0.90 indicates adequate fit and a value greater than or equal to 0.95 indicates very good fit), (II) the Bollen Index (BFI), which also takes values between 0 and 1 (values greater than 0. 90 are considered adequate and values greater than 0.95 are considered very good), (III) the McDonald index (with similar interpretations as CFI and IFI), and (IV) the root mean square error (RMSEA), which should have values less than or equal to 0.06 to indicate very good fit (20).

**Figure 1:**
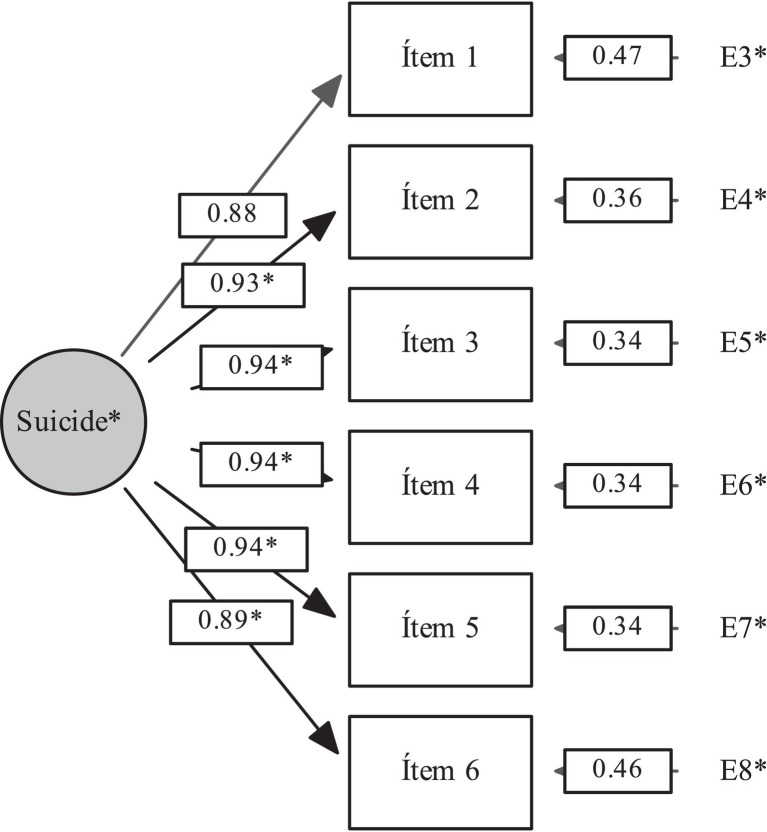
Confirmatory factor analysis of the C-SSRS suicide risk construct in the spanish online questionnaire. Chi-squared = 5010.10, *p* < 0.001, CFI = 0.99, RMSEA = 0.05.

We used an item response theory (IRT) model *via* a two-parameter model (“a” and “b”), in which “a” indicates the discrimination index, the ability of items to discriminate efficiently between at-risk and non-at-risk individuals, and “b” indicates the difficulty index when it comes to latent variables that measure performance. In this case, this index is interpreted as a measure of the relative position of the severity of suicide risk (21). The aim of this analysis was to confirm the theoretically proposed severity index, which is important evidence of validity (Figure 1). Psychometric analysis was performed using XCalibre 4.2.2 software (22), and CFA was performed using EQS 6.2 software (23).

## 3. Results

### 3.1. Reliability and dimensionality analysis

The initial analysis yielded a Cronbach’s alpha coefficient of 0.81. Table 2 shows the tetrachoric correlation matrix, which shows high correlations among the items. Figure 1 shows the high factor loadings (0.88–0.94) obtained in the CFA; this indicates high unidimensionality, indicating construct validity for the severity of suicide risk, which includes three specific attributes: Ideation, Planning, and Attempt. The indices showed good model fit (CFI = 0.995; IFI = 0.995, MFI = 0.990, and RMSEA = 0.047,95% CI [0.038–0.056]); this fit was achieved by all additional parameters.

**Table 2:** Tetrachoric correlation matrix.

	Item 1	Item 2	Item 3	Item 4	Item 5	Item 6
Item 1	1					
Item 2	0.934	1				
Item 3	0.859	0.896	1			
Item 4	0.781	0.853	0.899	1		
Item 5	0.802	0.856	0.866	0.9	1	
Item 6	0.685	0.782	0.797	0.878	0.916	1
*M*	0.1386	0.0873	0.04	0.0298	0.0285	0.0194
*SD*	0.3455	0.2824	0.1959	0.1702	0.1664	0.1381

### 3.2. Discrimination analysis and item difficulty

The results indicate that the proposed theoretical model was confirmed (Table 1). Column I of Table 3 shows the classical *p* and *R* indices, which represent the proportion of cases that answered the questions affirmatively and the biserial correlation between the answers and the total score of the questionnaire. Indices *a* and *b* correspond to the discrimination parameter and the position parameter calculated in IRT; parameter *b* in this case represents the severity of suicide risk. The discrimination values are generally between 0.4 and 1.5, and the values obtained indicate high discrimination power in all cases. The same table shows that the Z Resid and *p*-values are not significant, indicating that there are no significant differences between the theoretical and empirical models, so it can be assumed that the parameters are stable and able to efficiently discriminate individuals at higher risk.

**Table 3:** Values derived from the psychometric and severity analyses of the items.

	(I) Psychometric results of the test items						(II) Severity analysis	
Item	*p*	*R*	a	b	Z Resid	*p*	Severity	b
1	0.139	0.589	1.843	1.756	1.5325	0.1254	Low	1.756
2	0.087	0.716	2.890	2.118	1.648	0.0993	Low	2.118
3	0.040	0.658	1.664	2.889	0.7781	0.4365	Medium	2.889
4	0.030	0.620	1.492	3.175	0.6252	0.5318	Medium	3.175
5	0.028	0.620	1.505	3.206	0.6217	0.5342	High	3.206
6	0.019	0.501	1.161	3.704	0.5193	0.6035	High	3.704

Table 3, column II in shows each item and the empirically estimated severity. It also shows that the values of *b* increase gradually, as do the theoretical severities suggested by the judges, so this relationship provides evidence of content validity.

**Figure 2:**
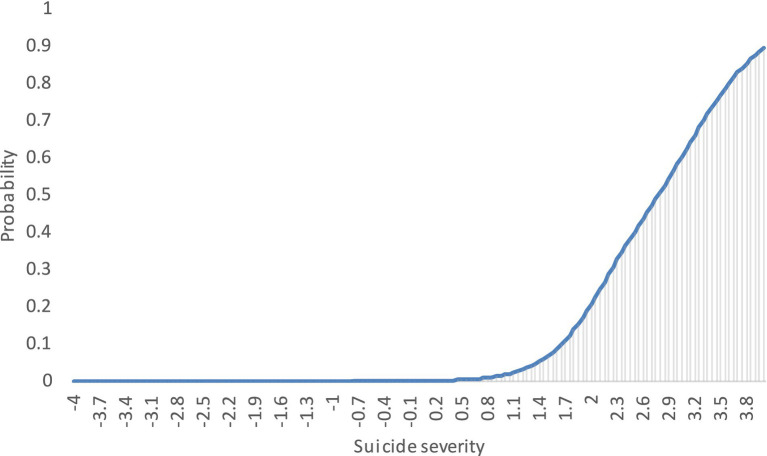
C-SSRS test response function.

Figure 2 shows the conditional relationship between the increase in symptoms and the probability of answering a larger number of items. The graph shows that as the severity of suicide risk increases (x-axis), the probability (y-axis) of subjects answering “yes” to the questions in the questionnaire increases. The lower the subjects’ suicide risk, the lower the probability that they will answer the questions in the affirmative. This indicates that the questionnaire is effective in distinguishing between highly suicidal and non-suicidal subjects.

## 4. Discussion

The results of the psychometric analyses conducted with this electronic Spanish-language version of the C-SSRS demonstrate a reasonable level of reliability, construct validity, and unidimensionality in the assessment of suicide risk. The analysis of the items indicates that they follow the proposed theoretical model and demonstrate the validity of the risk levels proposed in the six-item version, so we can assume that this version of the C-SSRS is able to efficiently discriminate individuals at higher suicide risk.

These results favor use of this scale and respond to some of the criticisms of its performance (24): its contribution to identifying severity is clear and the wording of the items was understandable to the Mexican participants. The verification of the unidimensionality of the construct is another important contribution, consistent with the findings of a meta-analysis (25), which showed that suicidal ideation and behaviors (understood as the presence of self-injury, attempt, or suicidal behavior) are moderately associated with suicide; that is, no conclusive evidence was found that suicidal behaviors are more strongly related to suicide than suicidal ideation. Thus, the recommendation to staff who provide clinical care and follow-up for people with suicidal behavior is not to privilege suicidal behavior over suicidal ideation, since the assessment of suicide risk is a unidimensional construct.

The results of this study also provide an opportunity to resolve some difficulties identified with the items, namely that the lower-level items (generally the items on suicidal ideation) captured less information about suicidal risk than the upper-level items (on planning and attempt), and that variation between individuals at the lower end of the scale was more error-prone than variation at the upper end. That is, items in the “once in a lifetime” time window (which is usually the first screening for all respondents) were the most problematic (14). This could be due to ambiguous wording leading to different interpretations and a large range of responses among respondents, such that the lower-level items provide less useful information about suicide risk than the upper-level items. Because the C-SSRS uses a conditional response design in which the higher-level items are presented only when the lower-level items are answered in the affirmative, the results suggest that the scale may lead to greater measurement error than expected when rating individuals (14). However, this is not the case in the version we present, as the questionnaire can be configured to answer all questions when used online.

### 4.1. Recommendations for the use of the scale

Some authors contend that the predictive value of suicide risk assessment tools is limited and counterproductive, primarily because classification as high, moderate, or low risk could be used to misallocate care by denying necessary treatment to some and providing unnecessary and restrictive treatment to others (26, 27), especially for those classified as high risk (28). With this in mind, it should be considered that as a first step toward timely and comprehensive suicidality care, public mental health services should use tools to identify high-risk individuals who are at imminent risk whether or not they report suicidal ideation (29), require more detailed assessment and follow-up, whether in the form of hospitalization or intensive support in the community (26). Thus, a shift from traditional risk stratification to a more clinically meaningful learning-based model is recommended (30). Such a model should be based on needs assessment rather than risk assessment and should allow the identification of the development, maintenance, and generalization of suicidal behavior, which would be useful for the assignment of subsequent clinical care aimed at achieving autonomy in individuals and thereby preventing suicides (31, 32). Similarly, the application of therapeutic approaches with clinical and scientific evidence for the assessment and management of suicide risk will allow effective support for suicidal individuals (33) and provide healthcare professionals with confidence in clinical practice (34). Along these lines, positive experiences have been documented with up to 35% reductions in suicide attempt recidivism after a shift to collaborative risk assessment that is more person-centered, along with safety planning, psychoeducation of at-risk individuals and their caregivers, and assertive follow-up (34, 35). This implies highlighting the importance of reforming suicide risk assessment practices in health services, which should not be based solely on the use of suicide risk screening.

Aspects of implementing a screening strategy must also be considered. These include the training and awareness needed to promote empathy and safety among field staff using the tool. It is known that comfort in initiating a suicide interview is greater when information is provided on how to help a person in this situation or when tools are used that provide guidance on questions to ask and strategies for proceeding (6). Other helpful measures include improving clinical education, improving the identification of at-risk individuals who visit a health care facility, developing clinical and safety pathways for patients who are considered at-risk, and increasing the availability of individuals who can serve as trusted contacts for individuals in suicide crisis (5).

Clinical decisions should be made with caution and should not be based solely on the severity of risk from the C-SSRS, because people with different scores may have similar suicide risk and people with the same score may have markedly different risk (14). Scores should be considered with caution. On the other hand, it is important to have scoring systems that are useful for clinical research in suicide risk. For example, clinical trials that seek to test the effect of interventions to reduce suicide risk need a measure that (a) accurately captures suicide risk, (b) is sensitive to change, (c) can distinguish between a therapeutic intervention and a placebo, and (d) has sufficient granularity so that a reduction in suicidal ideation can be translated into suicide risk (i.e., presence or absence of a plan and presence of suicide). In addition, the ideal instruments would reduce participant burden and study costs (36) and would be invariant for relevant variables such as age, gender, and schooling. This is revealing because in Mexico, adolescents are an at-risk group (under 18 years of age), and as in other parts of the world, suicide rates are higher among men (10.9 suicides per 100,000) than among women (2.4 per 100,000) (2), and 63.4 of reported deaths by suicide in 2019 occurred in the population with basic education (primary and secondary) (37).

### 4.2. Public mental health implications of appropriate measurement of suicide risk

Public mental health interventions target two main areas, prevention, and promotion, and are recommended for preferential targeting to groups at higher risk for mental disorders and distress over the general population (38, 39). Given the relative rarity of suicide deaths and the clinical and scientific challenges associated with screening, screening may not accurately identify individuals at risk (26). However, if screening tools are available, quick, easy to use, economically feasible, reliable, and valid, they may form the basis for prevention strategies that could focus on combining universal interventions with selective and indicated interventions that consider identification of high-risk individuals and assessment and evaluation for more specific psychological or psychiatric interventions.

Real-time monitoring of specific groups could also be used to reach different geographic areas and obtain differentiated snapshots for targeted and localized actions (27). Currently, there is more reliance on statistical surveys, which do not provide the same opportunities for a timely and tailored response (3).

The promotion of strategies aimed at reducing exposure to modifiable risk factors is essential to the provision of effective interventions for selected subpopulations and for unselected clinical populations (40). Thus, care and treatment should be provided not only in clinical settings, because there are other variables (sex, age, sociodemographic context) that are more likely to be related to access to formal general health and mental health services than to death by suicide. Evidence shows that many people who die by suicide did not have access to needed mental health care, did not report previous suicidal behavior, and their methods were more likely to be lethal, so screening tools in different settings might be a good strategy, especially among at-risk groups (33).

It should be noted that suicide risk assessment is not the same as risk management, so mere assessment without the development of a management plan according to the magnitude and nature of the risk is unlikely to improve outcomes for individuals; therefore, risk scales should not replace comprehensive psychosocial assessment (31). The goal of mental health policy should be to move mental health out of its current professional, organizational, and even political isolation and place it within a broader framework, that is, to shift the focus from the individual level to strengthening the population mental health approach (41). Public health approaches to suicide prevention must incorporate social and cultural frameworks to develop strategies that save the most lives in an effective and measurable way (41). Selective prevention strategies that focus on high-risk groups is important from an ethical perspective because it could reduce the suffering of individuals and their families. Its combination with universal approaches could help prevent a greater number of deaths (41).

### 4.3. Limitations

Our study includes limitations inherent in the design and nature of the sample, since the population that participated in the online questionnaire was the one that learned about the survey and had access to electronic devices and an internet connection to answer the survey, however, in Mexico, the percentage of internet access is 70.1% (42). On the other hand, there is only one measurement, it is not possible to assess predictive power, but it is important to note that given the dynamic nature of suicide risk, the focus of the assessment should be on modifiable factors and safety planning rather than just predicting risk (26).

Finally, it should be noted that the version analyzed does not investigate whether a suicide attempt is currently being considered, which is essential for identifying the at-risk population. A question to this effect should be added in future applications of the scale, as well as in different Spanish-speaking populations and in different application modalities.

## 5. Conclusion

The six-item Spanish online version of the C-SSRS showed adequate psychometric properties in a sample of the Mexican population. Although we believe that a risk assessment tool is not a substitute for a clinical approach, it is a tool that helps to identify the population at risk and refer them to care according to the level of risk identified. The assessment is fundamental in determining a person’s level of risk and influences the way the case is approached, helping health care professionals make decisions to prevent death by suicide and contribute to building a meaningful life for the person at risk.

## Data availability statement

The raw data supporting the conclusions of this article will be made available by the authors, without undue reservation.

## Ethics statement

The studies involving humans were approved by Research Ethics Committee of the Instituto Nacional de Psiquiatría Ramón de la Fuente Muñiz (study no. CEI/C/059/2020). The studies were conducted in accordance with the local legislation and institutional requirements. The participants provided their written informed consent to participate in this study.

## Author contributions

AJ-T, CA-G, JR, and IG: Conceptualization and methodology. FA-C: formal analysis. FA-C, AJ-T, and CA-G: research and writing of original draft. CA-G and AJ-T: data curation. All authors contributed to the article and approved the submitted version.

## Funding

This research was partially funded by the Icahn School of Medicine at Mount Sinai.

## Conflict of interest

The authors declare that the research was conducted in the absence of any commercial or financial relationships that could be construed as a potential conflict of interest.

## Publisher’s note

All claims expressed in this article are solely those of the authors and do not necessarily represent those of their affiliated organizations, or those of the publisher, the editors and the reviewers. Any product that may be evaluated in this article, or claim that may be made by its manufacturer, is not guaranteed or endorsed by the publisher.
