# Supplementary material for: The Columbia-suicide severity rating scale: validity and psychometric properties of an online Spanish-language version in a Mexican population sample
Source: Front Public Health. 2023 Sep 5;11:1157581. doi: 10.3389/fpubh.2023.1157581 (PMC10507718; doi:10.3389/fpubh.2023.1157581)
Supplement: Supplementary file 1 [file Table_1.docx]

Supplementary Material

The Columbia-Suicide Severity Rating Scale (C-SSRS): Validity and Psychometric Properties of an Online Spanish-language Version in a Mexican Population Sample

**Supplementary Table 1.** Columbia-Suicide Severity Rating Scale (C-SSRS) items in Spanish for online application.

|  | **Mes pasado** | |
| --- | --- | --- |
| **Responda todas las preguntas** | **Sí** | **No** |
| 1. ¿Ha pensado (aunque sea por un momento) que estaría mejor muerto/muerta, ha deseado morir o ha sentido que necesitaba morir? |  |  |
| 2. ¿Ha pensado (aunque sea por un momento) en dañarse, lastimarse o lesionarse a usted mismo/misma con la intención o la seguridad de que podría morir? |  |  |
| 3. ¿Ha hecho un plan (por ejemplo, escoger un lugar, una fecha, una época) para intentar suicidarse? |  |  |
| 4. ¿Ha hecho algunas cosas para preparar un intento de suicidio con el que esperaba o intentaba morir? |  |  |
| 5. ¿Ha comenzado un intento de suicidio, pero decidió detenerse y no lo completó? |  |  |
| 6. ¿Ha comenzado un intento de suicidio, pero alguien lo/la interrumpió y no pudo completarlo? |  |  |
|  | | |

Riesgo bajo

Riesgo moderado

Riesgo alto
